# Fast and accurate electromagnetic field calculation for substrate-supported metasurfaces using the discrete dipole approximation

**DOI:** 10.1515/nanoph-2023-0423

**Published:** 2023-10-23

**Authors:** Weilin Liu, Euan McLeod

**Affiliations:** Wyant College of Optical Sciences, University of Arizona, 1630 E University Blvd, Tucson, AZ 85719, USA

**Keywords:** metasurface, discrete dipole approximation, finite difference time domain, Sommerfeld integral, cylindrical Green’s function

## Abstract

Metasurface design tends to be tedious and time-consuming based on sweeping geometric parameters. Common numerical simulation techniques are slow for large areas, ultra-fine grids, and/or three-dimensional simulations. Simulation time can be reduced by combining the principle of the discrete dipole approximation (DDA) with analytical solutions for light scattered by a dipole near a flat surface. The DDA has rarely been used in metasurface design, and comprehensive benchmarking comparisons are lacking. Here, we compare the accuracy and speed of three DDA methods—substrate discretization, two-dimensional Cartesian Green’s functions, and one-dimensional (1D) cylindrical Green’s functions—against the finite difference time domain (FDTD) method. We find that the 1D cylindrical approach performs best. For example, the *s*-polarized field scattered from a silica-substrate-supported 600 × 180 × 60 nm gold elliptic nanocylinder discretized into 642 dipoles is computed with 0.78 % pattern error and 6.54 % net power error within 294 s, which is 6 times faster than FDTD. Our 1D cylindrical approach takes advantage of parallel processing and also gives transmitted field solutions, which, to the best of our knowledge, is not found in existing tools. We also examine the differences among four polarizability models: Clausius–Mossotti, radiation reaction, lattice dispersion relation, and digitized Green’s function, finding that the radiation reaction dipole model performs best in terms of pattern error, while the digitized Green’s function has the lowest power error.

## 1 Introduction

Metasurfaces, as two-dimensional (2D) metamaterials, use engineered nanostructures to manipulate the propagation direction and polarization of light in a form factor that is much thinner and lighter than conventional refractive and diffractive optics [1]. The high refractive index contrast in metasurface unit cells renders stronger subwavelength light confinement and light–matter interaction than conventional optics, empowering many new spectral and polarization functionalities in applications like perfect absorbers [2], low aberration metalenses [3], compact beam splitters [4], and polarization converters [5]. Moreover, compared with multilevel diffractive optics, the binary nanostructures in metasurfaces make them compatible with standard CMOS fabrication processes.

Metasurface design is tedious and time-consuming since it involves tailoring and positioning thousands of nanoantennas, or more. The design often involves iterative optimization methods. Reducing the number of iterations is a common option to shorten the computational time, using efficient optimization algorithms such as genetic algorithms [6] or particle swarm optimization [7] to rapidly escape local optima in favor of global optima. Dimensionality reduction [8] in neural networks using physical intuition [9], principal component analysis [10], or autoencoders [11] can reduce computational time by identifying the subset of design parameters that have the highest impact. Alternatively, design time can be reduced by reducing the computational time of individual simulations. A common simulation tool used in metasurface design is the finite difference time domain (FDTD) method, which was proposed by Yee [12] in 1966. Because of its versatile geometric modeling properties, it has been widely applied in photonic component design such as photovoltaics [13], photonic crystals [14], optical microrings, and resonators [15]. As a time-iterative method, FDTD can compute the response to broadband sources in a single simulation. However, simulations often take tens of minutes to even a few days depending on the number of grid points in the whole simulation region. Furthermore, most commercial FDTD solvers are relatively expensive.

The discrete dipole approximation (DDA), also called the coupled dipole method, can be applied to particle scattering and absorption computation for arbitrary particle composition and geometry. Structures with substantial volume need to be discretized into smaller “particles” to satisfy the dipole approximation. Developed and popularized by Draine and Flatau [16] in 1994, associated with an open source Fortran code DDSCAT, this method can provide high accuracy with relatively low computational cost, especially for particles with relative permittivity |*ɛ*| < 2. Initially used in applications such as optical tweezers [17], aerosol physics [18, 19], and surface-enhanced Raman scattering (SERS) [20], DDA was used for calculations for particles surrounded by a homogeneous medium. However, there are numerous applications like metasurfaces that require rigorous calculations for particles near a single or multilayered substrate. The substrate could be handled by brute-force via discretizing it into many dipoles. Although straightforward, this introduces an undesirable tradeoff: modeling a large substrate area and depth with many dipoles provides accuracy but with severe computational cost. If the dipoles are all placed on a regular array, then the dipole moment calculation can be accelerated using fast Fourier transforms (FFTs) [16], which makes the DDA calculation tractable for *N* ∼ 10^6^ particles, or more. But overall, brute-force discretization of the substrate is not an efficient approach.

In principle, it can be more efficient to analytically account for how the substrate affects the dipole coupling. This analytical solution under cylindrical coordinates was derived by Taubenblatt [21] in 1993 and then adapted to Cartesian coordinates by Schmehl [22] in 1997. By decomposing the spherical wave radiated from a dipole into cylindrical components, reflections off the substrate can be modeled using Fresnel coefficients. Despite its potential advantages in terms of efficiency, this method has rarely been used because of the difficulties in numerical integration induced by dipole–dipole interaction and the difficulties in applying FFT acceleration for dipole moment calculation. Several approximations were proposed to simplify calculations, such as placing image dipoles on the opposite side of the interface [23, 24], evaluating far-field Green’s functions by the stationary phase approximation [25], or approximating the Sommerfeld integrand (see Section 2.4) by a series of exponential functions [26]. However, the strict assumptions associated with these approximations limited their applicability for most applications. For example, the image dipole approximation is only valid for dipoles under the quasi-electrostatic limit (*k* → 0), so the retardation between dipoles with long separation distance is neglected as the phase term e^i ***kr***^ → 1. This approximation can provide fairly good accuracy for small dipoles with short range interactions in upper half-space.

An open source code called DDA-SI with complete and detailed derivation based on Taubenblatt’s paper was proposed in 2010 by Loke [27–29]. The computation of the electromagnetic field of the particles near the substrate is sophisticated because it involves Sommerfeld integration and second-order partial derivatives under cylindrical coordinates, but this approach offers highly accurate results. In 1997, Lukas Novotny [30] proposed an analytical electromagnetic field solution for both horizontally and vertically oriented dipoles located above a layered substrate based on the Hertz vector with simpler expressions that eliminate the partial derivatives in Taubenblatt’s and Schmehl’s methods. Importantly, solutions are valid for both the upper and lower spaces, making the implementation of the DDA method in metasurface design possible for both reflective and transmissive metasurfaces. More details about various DDA computational tools, such as ADDA [23, 31] and MPDDA [32], can be found in the review article [33].

In this paper, we compare the accuracy and computation speed between two-dimensional (2D) and one-dimensional (1D) methods of computing the Sommerfeld integrals for multiple dipoles. Our approach takes advantage of parallel processing for faster calculations and outputs both the transmitted and reflected fields, which, to the best of our knowledge, is not done in other existing simulation tools. We also evaluate the speed and accuracy of four different polarizability models: the simple Clausius–Mossotti relation, the radiation reaction correction, the lattice dispersion relation, and the digitized Green’s function. Using our 1D integration method and the radiation reaction correction dipole model, we find highly accurate and rapid near-field and scattered field calculation for light incident on elliptical cylinder (“pillbox”) shaped nanoparticles that are sitting on a substrate. This holds true for metallic and high-index dielectric particles and/or substrates. A benefit of our approach over FDTD is that the far-field calculation does not suffer from aperturing artifacts due to a small near-field computational domain and does not require any approximations, providing advantages for several different types of systems, such as the exploration of weak coupling among distant nanoparticles, as well as accurate far-field scattering patterns over large simulation regions.

Since our DDA-with-substrate method can rigorously calculate near- and far-fields, it is well suited for engineering nanostructures of any shape and composition on substrates. In the future, this approach can be used for creating a nanostructure library for metasurface design. Because metasurface unit cells are typically smaller than the wavelength, significantly fewer $\left(∼10^{4}\right)$ dipoles are required for accurate simulation than in many previous DDA applications. As a result, the electromagnetic field over a large monitor area can be obtained in the order of 1–10^3^ s, which can enable fast metasurface forward and inverse design and fast data set generation for machine learning.

## 2 Methods

### 2.1 System geometry

**Figure 1:**
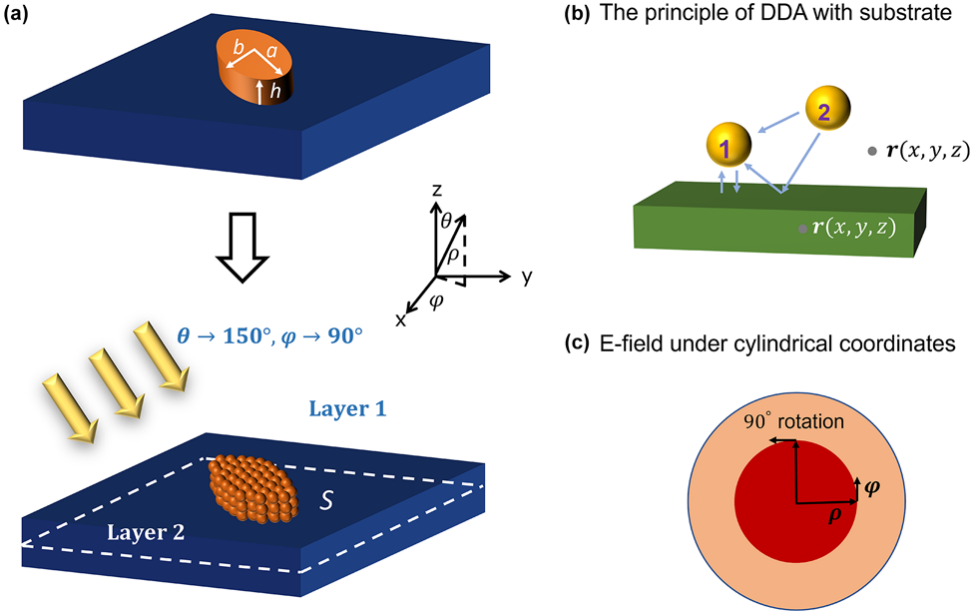
System geometry. (a) Structures on a substrate can be discretized into smaller, dipole-scale particles. To ensure our approach is generally applicable, symmetry is broken by choosing to simulate a rotated elliptic cylinder illuminated with an oblique plane wave. Two sizes of elliptic cylinder are simulated, as noted in the dimensions in panel (a). The incident wavelength is 1064 nm. The measurement plane is surface *S*, located 200 nm below the interface. (b) In the presence of a substrate, the DDA method is complicated by accounting for reflections off the substrate when computing interparticle coupling. (c) Diagram of the cylindrical coordinate system.

We use an elliptic cylinder seated on a substrate, as illustrated in Figure 1, to compare the accuracy and speed of various DDA methods to a reference FDTD approach. Either s or p polarized light with 1064 nm wavelength impinges on the structure with incidence angle *θ* = 150°, *φ* = 90°. A wavelength of 1064 nm was chosen because it is one of the most common laser wavelengths, and we have such a laser in our laboratory that we plan to use for future experiments. We collected the electric field 200 nm beneath the interface over a 2 μm × 2 μm area. Most of our results are for an elliptic cylinder with dimensions: long axis *a* = 300 nm, short axis *b* = 90 nm, height *h* = 60 nm, and the short axis is oriented along *ϕ* = 150°. For simulations involving a high-index substrate, we use a quarter-size elliptic cylinder with dimensions: *a* = 75 nm, *b* = 22.5 nm, and *h* = 15 nm, which allows us to test finer dipole discretizations without needing more computational memory. We test two materials for the elliptic cylinder: gold [34] with refractive index 0.1044 + 6.8635*i* and germanium [35] with index 4.3848 + 0.1513*i*. We also test two substrate materials: silica [36] with index 1.4496 and silicon [37] with index 3.5548.

For the DDA approaches, the elliptic cylinder is discretized into individual dipoles with a pitch *h*/*D*, where *D* is a parameter we call the discretizing factor. The dipoles are uniformly distributed, so a larger *D* means more dipoles across all dimensions of the elliptic cylinder.

### 2.2 FDTD

FDTD is a popular nanophotonic simulation method due to its versatility and accuracy, which is a consequence of its ability to directly solve Maxwell’s equations. However, the time-iterated solver can be computationally inefficient for single-frequency simulations. In addition, the need for a gridded modeling region with cell size significantly smaller than the wavelength makes the simulation of large scale structures intractable. Furthermore, numerical artifacts can sometimes cause unstable and erroneous results. Errors can be minimized by choosing proper grid sizes and time steps. To ensure that our FDTD simulations serve as an accurate reference for testing our DDA approach, we used an underlying coarse grid with automatic nonuniform mesh accuracy setting of 2 for the outer regions of the simulation domain. We also applied a finer grid to the entire simulation region to avoid large numerical dispersion, and we tested different grid sizes (20 nm, 10 nm, 5 nm, and 3.5 nm), which approximately correspond to (*λ*/50, *λ*/100, *λ*/200, and *λ*/300). We found convergence for all materials at 3.5 nm grid size, with the worst case being for gold particles, where there was a 4 % difference in the maximum electric field along *y*-direction between the 3.5 nm and 5 nm grid sizes.

The auto-shut off values and time steps are set to 10^−6^ and 0.99, respectively, to assure convergence and accuracy.

### 2.3 Free-space DDA formulation

The scattering and absorption of electromagnetic waves by particles of arbitrary shape and composition can be simulated by the DDA method. Modeling this interaction in free space forms the basis for the model involving a substrate. When the wavelength is much larger than the particle size, the *j*th particle can be approximated as a single dipole with dipole moment,

(1) $$p_{j}=\varepsilon_{b}\alpha_{j}E_{j}.$$

where *ɛ*_*b*_ is the background relative permittivity, **E**_*j*_ is the electric field at dipole location **r**_*j*_, and *α*_*j*_ is the polarizability of the dipole. For nonlinear processes, such as those in references [38, 39], higher order terms would also need to be included here. Polarizability can depend on particle shape, size, and material. There are various dipole models used for computing polarizabilities. In this paper, we will discuss four different dipole models based on Clausius–Mossotti relation, radiation reaction correction, digitized Green’s function, and lattice dispersion relation and how these polarizabilities affect the simulation results in Section 3.2. When not otherwise noted, we use the radiation reaction polarizability due to its high performance. The driving field for dipole *j* is the superposition of the incident field evaluated at **r**_*j*_ and the scattered field from the other *N* − 1 particles with dipole moments **p**_*k*_ at locations **r**_*k*_,

(2) $$E_{j}=E_{\text{inc},j}+\frac{\omega^{2}}{\varepsilon_{0}c^{2}}\underset{k\neq j}{\sum }{\overset{⃡}{G}}_{jk}^{\text{FS}}p_{k},$$

where *ω* is the frequency of incident light, *c* denotes the speed of light, *ɛ*_0_ is the vacuum permittivity, **E**_inc,*j*_ is the incident field at the center of particle *j*, and ${\overset{⃡}{G}}_{jk}^{\text{FS}}={\overset{⃡}{G}}^{\text{FS}}\left(r_{j},r_{k}\right)$ is given by the dyadic Green’s function in free-space [40]:

(3) $$\begin{array}{ll} {\overset{⃡}{G}}^{\text{FS}}\left(r,r_{0}\right) & =\frac{e^{\mathrm{ikR}}}{4\pi R}\left[\left(1+\frac{ikR-1}{k^{2}R^{2}}\right)\overset{⃡}{I}\right.\\ & \;\left.+\frac{3-3ikR-k^{2}R^{2}}{k^{2}R^{2}}\frac{R⊗R}{R^{2}}\right], \end{array}$$

where **r** is the evaluation point, **r**_**0**_ is the dipole location, **R** = **r** − **r**_0_, *R* = |**R**|, *k* (when not used as a subscript) is the magnitude of the wave vector **k**, $\overset{⃡}{I}$ is the 3 × 3 identity matrix, and ⊗ represents the outer (tensor) product of two vectors. For the special case *j* = *k*, we define ${\overset{⃡}{G}}_{jj}^{\text{FS}}=-\frac{\varepsilon_{0}c^{2}}{\omega^{2}\varepsilon_{b}\alpha_{j}}$ . Using Equation (1), **E**_*j*_ can be written as $E_{j}=\frac{p_{j}}{\varepsilon_{b}\alpha_{j}}=-\frac{\omega^{2}}{\varepsilon_{0}c^{2}}{\overset{⃡}{G}}_{jj}^{\text{FS}}p_{j}$ , so that Equation (2) can be reformulated to include **E**_*j*_ in the sum:

(4) $$\overset{N}{\underset{k=1}{\sum }}{\overset{⃡}{G}}_{jk}^{\text{FS}}p_{k}=-\frac{\varepsilon_{0}c^{2}}{\omega^{2}}E_{\text{inc},j},$$

which is a set of 3*N* linear equations for each of the components of the dipole moments **p**_*k*_. Once the dipole moments are known, the total electric field can be calculated at an arbitrary location **r** by,

(5) $$E\left(r\right)=E_{\text{inc}}\left(r\right)+\frac{\omega^{2}}{\varepsilon_{0}c^{2}}\overset{N}{\underset{k=1}{\sum }}{\overset{⃡}{G}}^{\text{FS}}\left(r,r_{k}\right)p_{k}.$$

A substrate can be incorporated into the free-space DDA formulation by discretizing the substrate into many dipoles. Here, we use a dipole spacing of 200 nm, distributed over an area of 9.8 μm × 9.8 μm and a depth of 1 μm. We compare the performance (speed and accuracy) of this computational approach to FDTD and the following two alternative DDA approaches. In the next formulation, reflections are calculated via two-dimensional Sommerfeld integrals in Cartesian coordinates, while in the final formulation, reflections are calculated via one-dimensional integrals in cylindrical coordinates.

### 2.4 Cartesian Green’s function: 2D integration

In this method, originally derived by W. Lukosz and R. E. Kunz in 1977 [30, 40], the light reflected by and/or transmitted through the substrate from a dipole source is analytically calculated, and these contributions are included as additional 2D Green’s functions, depicted by the blue arrows in Figure 1(b). Analogously to Equation (5), provided that the dipole moments are known, the electric field at an arbitrary location **r** can be calculated by [40],

(6) $$E\left(r\right)=\left\{\begin{array}{c} \frac{\omega^{2}}{\varepsilon_{0}c^{2}}\overset{N}{\underset{k=1}{\sum }}\left[{\overset{⃡}{G}}^{\text{FS}}\left(r,r_{k}\right)+{\overset{⃡}{G}}^{\text{ref}}\left(r,r_{k}\right)\right]p_{k}+\;\\ E_{\text{inc}}\left(r\right)+E_{\text{inc}-\text{ref}}\left(r\right),\;\;\;\;\;\;\;\;\;\;\;\;\;\;\;\;\;\;\;\;\;\;\;\;\;\;\;\;\;\;\;z>0\;\\ E_{\text{inc}-\text{tra}}\left(r\right)+\frac{\omega^{2}}{\varepsilon_{0}c^{2}}\overset{N}{\underset{k=1}{\sum }}{\overset{⃡}{G}}^{\text{tra}}\left(r,r_{k}\right)p_{k},\;\;\;\;\;\;\;\;\;\;z<0\; \end{array}\right.$$

where *z* > 0 corresponds to the background area (incident medium), and *z* < 0 corresponds to the substrate, as indicated in Figure 1, **E**_inc-ref_ and **E**_inc-tra_ are the reflected and transmitted incident fields calculated based on the Fresnel coefficients and Snell’s law (see Appendix A), ${\overset{⃡}{G}}^{\text{tra}}$ is the Green’s function in the substrate (Appendix A), and ${\overset{⃡}{G}}^{\text{ref}}$ is the contribution from the electric field reflected off of the substrate [40]:

(7) $$\begin{array}{ll} {\overset{⃡}{G}}^{\text{ref}}\left(r,r_{0}\right) & =\frac{i}{8\pi^{2}}\overset{\infty }{\underset{-\infty }{\iint }}\left[{\overset{⃡}{M}}_{\text{ref}}^{s}\left(k_{x},k_{y}\right)+{\overset{⃡}{M}}_{\text{ref}}^{p}\left(k_{x},k_{y}\right)\right]\\ & \;\times e^{\text{i}\left[k_{x}\left(x-x_{0}\right)+k_{y}\left(y-y_{0}\right)+k_{1z}\left(z+z_{0}\right)\right]}\;dk_{x}\;dk_{y}. \end{array}$$

This calculation of the reflected Green’s function involves an integral over the angular spectrum of the field, as well as separate terms for s and p polarizations. The *z*-component of the wavevector is dependent upon the other two components according to, $k_{nz}=\pm \sqrt{k_{n}^{2}-k_{x}^{2}-k_{y}^{2}}$ , where *n* = 1, 2 indexes the half-space and the sign of the root is chosen such that Im(*k*_*nz*_) ≥ 0. The expressions for ${\overset{⃡}{M}}_{\text{ref}}^{s}$ and ${\overset{⃡}{M}}_{\text{ref}}^{p}$ are given in Appendix A and involve singularities at particular values of (*k*_*x*_, *k*_*y*_). The integration makes the DDA-substrate problem significantly more computationally challenging than the free-space problem, for which there is an explicit analytical Green’s function (Equation (3)).

The DDA problem analogous to Equation (4) becomes,

(8) $$\sum_{k=1}^{N}\left[{\overset{⃡}{G}}_{jk}^{\text{FS}}+{\overset{⃡}{G}}_{jk}^{\text{ref}}\right]p_{k}=-\frac{\varepsilon_{0}c^{2}}{\omega^{2}}\left[E_{\text{inc},j}+E_{\text{inc}-\text{ref},j}\right].$$

Although relatively straightforward, this 2D Green’s function approach has seen little application, principally because the Sommerfeld integrals in Equation (7) are difficult to evaluate numerically due to the infinite range of integration, high-frequency oscillatory behavior for large *k*_*x*_ and *k*_*y*_, and an infinite number of singular points in ${\overset{⃡}{M}}_{\text{ref}}^{s}$ and ${\overset{⃡}{M}}_{\text{ref}}^{p}$ . More detailed discussions about Sommerfeld integrals can be found in [41].

The reflection and transmission dyadics ${\overset{⃡}{M}}_{\text{ref}}^{s}$ , ${\overset{⃡}{M}}_{\text{ref}}^{p}$ , ${\overset{⃡}{M}}_{\text{tra}}^{p}$ , and ${\overset{⃡}{M}}_{\text{tra}}^{s}$ can be numerically approximated by using the built-in Matlab function trapz, which performs trapezoidal integration. Sampling in frequency space is defined by d*k*_*x*_ and d*k*_*y*_, and the infinite integration intervals are truncated to {−*k*_max_ < *k*_*x*_ < *k*_max_, −*k*_max_ < *k*_*y*_ < *k*_max_}. However, this method does not handle singularities well, with a typical approach of replacing any nonfinite integrand values with 0. Also, the choice of proper *k*_max_ depends on the *z*-coordinate of each dipole. For example, for the materials and dipole spacing that we use here, when dipoles are located 2 μm above the substrate surface, *k*_max_ = 3*k*_1_ with 104 × 106 sampling points in frequency space gives acceptable results, while for dipoles less than 20 nm from the substrate, *k*_max_ = 250*k*_1_ with 3104 × 3106 sampling points are necessary. More details can be found in Appendix B.

### 2.5 Cylindrical Green’s function: 1D integration

We derive the cylindrical Green’s functions for a dipole above a substrate by starting from the cylindrical coordinate (*ρ*, *φ*, *z*) expressions for the electric field scattered by a single dipole located at $r_{0}={\left(0,0,z_{0}\right)}^{T}$ . First, we calculate the scattered fields from a vertically (*V*) polarized dipole and an *x*-polarized horizontal (*H*) dipole [40, 42]. The scattered field from an arbitrary dipole moment $p={\left(p_{x},p_{y},p_{z}\right)}^{T}$ at any location above the substrate can be found by rotating, translating, and superimposing the fundamental *V* and *H* solutions. There are 12 fundamental electric field solutions: 3 polarization components (*E*_*ρ*_, *E*_*φ*_, *E*_*z*_) in the incident (*n* = 1) and substrate (*n* = 2) layers, for each of the *V* and *H* dipoles. To illustrate the form of these solutions, we show two here; the others can be found in Appendix C:

(9) $$\begin{array}{ll} E_{1\rho }^{V} & =p_{z}{\tilde{E}}_{1\rho }^{V}\\ & =\rho \left(z-z_{0}\right)\frac{p_{z}}{4\pi \varepsilon_{0}\varepsilon_{1}}\frac{e^{\text{i}k_{1}R}}{R^{3}}\left[\frac{3}{R^{2}}-\frac{3ik_{1}}{R}-k_{1}^{2}\right]\\ & \;-\frac{ip_{z}}{4\pi \varepsilon_{0}\varepsilon_{1}}\overset{\infty }{\underset{0}{\int }}J_{1}\left(k_{\rho }\rho \right)A_{1}k_{\rho }k_{1z}e^{\text{i}k_{1z}\left(z+z_{0}\right)}dk_{\rho }, \end{array}$$

(10) $$\begin{array}{ll} E_{2\varphi }^{H} & =\sin\varphi \;p_{x}\;{\tilde{E}}_{2\varphi }^{H}\\ & =\sin\varphi \frac{p_{x}}{4\pi \varepsilon_{0}\varepsilon_{1}}\overset{\infty }{\underset{0}{\int }}e^{\text{i}\left(k_{1z}z_{0}-k_{2z}z\right)}\left\{\frac{1}{\rho }J_{1}\left(k_{\rho }\rho \right)\right.\\ & \left.\left[k_{\rho }B_{2}+ik_{2z}C_{2}\right]-k_{2}^{2}J_{0}\left(k_{\rho }\rho \right)B_{2}\right\}dk_{\rho }, \end{array}$$

where *R* is the distance between the dipole and observation point, as in Equation (3). Note that the *φ* and dipole moment dependence can be easily separated out of these expressions with $\tilde{E}$ defined as the *φ*-independent and dipole moment-independent part of the expression. The wavenumbers in the incident and substrate layers are given by *k*_1_ and *k*_2_, respectively, while *k*_*ρ*_ is the radial part of the wavevector, and the axial part of the wavevector is determined by $k_{nz}=\sqrt{k_{n}^{2}-k_{\rho }^{2}}$ , for *n* = 1, 2. The coefficients *A*_*n*_, *B*_*n*_, and *C*_*n*_ in Equations (9) and (10) are related to the Fresnel coefficients and their expressions can be found in Appendix C. Note that the expression for $E_{1\rho }^{V}$ has a nonintegral term that corresponds to the free-space scattered field and an integral term that corresponds to the reflected field, whereas the expression for $E_{2\varphi }^{H}$ only has an integral term for the transmitted field.

A *y*-polarized dipole would have a scattered field that is a 90° counterclockwise rotation of the *x*-polarized dipole, as illustrated in Figure 1(c):

(11) $$E_{n}^{y}\left(\rho ,\varphi ,z\right)=E_{n}^{H}\left(\rho ,\varphi -\frac{\pi }{2},z\right).$$

Since the polarization components are expressed in cylindrical coordinates in the above equation, no rotation in polarization is necessary in mapping the field from an *x*-polarized horizontal dipole to that of a *y*-polarized dipole. The electric field polarization components expressed in cylindrical coordinates can be converted to Cartesian components via multiplication with the rotation matrix,

(12) $$\overset{⃡}{\Phi }\left(\varphi \right)=\left[\begin{array}{ccc} \cos\varphi & -\sin\varphi & 0\\ \sin\varphi & \cos\varphi & 0\\ 0 & 0 & 1 \end{array}\right].$$

Derived from the full expressions for the electric fields (Equations (9) and (10) and Appendix C), the scattered fields from *x*, *y*, and *z*-oriented dipoles can be concisely expressed using Green’s functions:

(13) $$\begin{array}{ll} \left[\begin{array}{c} E_{x}\\ E_{y}\\ E_{z} \end{array}\right] & =\overset{⃡}{\Phi }\left[\begin{array}{c} E_{n\rho }\\ E_{n\varphi }\\ E_{nz} \end{array}\right]\\ & =\overset{⃡}{\Phi }\left[\begin{array}{ccc} \cos\varphi {\tilde{E}}_{n\rho }^{H} & \cos\left(\varphi -\pi /2\right){\tilde{E}}_{n\rho }^{H} & {\tilde{E}}_{n\rho }^{V}\\ \sin\varphi {\tilde{E}}_{n\varphi }^{H} & \sin\left(\varphi -\pi /2\right){\tilde{E}}_{n\varphi }^{H} & {\tilde{E}}_{n\varphi }^{V}\\ \cos\varphi {\tilde{E}}_{nz}^{H} & \cos\left(\varphi -\pi /2\right){\tilde{E}}_{nz}^{H} & {\tilde{E}}_{nz}^{V} \end{array}\right]\left[\begin{array}{c} p_{x}\\ p_{y}\\ p_{z} \end{array}\right]\\ & =\overset{⃡}{\Phi }\left[\begin{array}{ccc} \cos\varphi {\tilde{E}}_{n\rho }^{H} & \sin\varphi {\tilde{E}}_{n\rho }^{H} & {\tilde{E}}_{n\rho }^{V}\\ \sin\varphi {\tilde{E}}_{n\varphi }^{H} & -\cos\varphi {\tilde{E}}_{n\varphi }^{H} & 0\\ \cos\varphi {\tilde{E}}_{nz}^{H} & \sin\varphi {\tilde{E}}_{nz}^{H} & {\tilde{E}}_{nz}^{V} \end{array}\right]p\\ & =\left[\begin{array}{ccc} {\tilde{E}}_{n\rho }^{H}\cos^{2}\varphi -{\tilde{E}}_{n\varphi }^{H}\sin^{2}\varphi & \left({\tilde{E}}_{n\rho }^{H}+{\tilde{E}}_{n\varphi }^{H}\right)\sin\varphi \cos\varphi & {\tilde{E}}_{n\rho }^{V}\cos\varphi \\ \left({\tilde{E}}_{n\rho }^{H}+{\tilde{E}}_{n\varphi }^{H}\right)\sin\varphi \cos\varphi & {\tilde{E}}_{n\rho }^{H}\sin^{2}\varphi -{\tilde{E}}_{n\varphi }^{H}\cos^{2}\varphi & {\tilde{E}}_{n\rho }^{V}\sin\varphi \\ {\tilde{E}}_{nz}^{H}\cos\varphi & {\tilde{E}}_{nz}^{H}\sin\varphi & {\tilde{E}}_{nz}^{V} \end{array}\right]p\\ & =\frac{\omega^{2}}{\varepsilon_{0}c^{2}}{\overset{⃡}{G}}^{1\text{D},n}p. \end{array}$$

**Figure 2:**
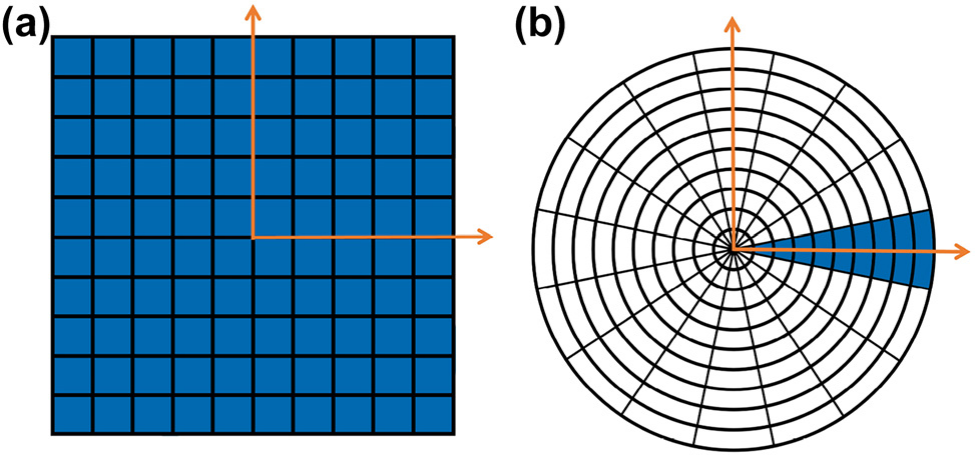
Computational advantage of the 1D Green’s function over the 2D Green’s function. (a) Following the 2D Green’s function approach, the field over an area would have to be calculated at each pixel location. (b) Following the 1D approach, the slow computational step of Sommerfeld integral calculation, e.g., Equation (7), need only be calculated over one radial line and then mapped (interpolated) across the whole area by rapid multiplications with trigonometric functions of *φ*.

Equation (13) can be considered a definition for ${\overset{⃡}{G}}^{1D,n}$ , which only involves 1D integrals. With this 1D approach, the computationally costly calculation of the Sommerfeld integral calculation, e.g., Equations (9) and (10), need only be calculated over one radial line and then mapped (interpolated) across the whole 2D area (Figure 2).

In the incident layer, these Green’s functions can be partitioned into a free-space part and a reflected part corresponding to the nonintegral and integral terms in the electric field expressions (e.g., Equations (9) and (10)): ${\overset{⃡}{G}}^{1\text{D,}1}={\overset{⃡}{G}}^{\text{FS}}+{\overset{⃡}{G}}^{1\text{D,ref}}$ . For computational simplicity, we use the inherently Cartesian expression (Equation (3)) for the free-space contribution and only apply the manipulations shown in Equation (13) to compute the reflected and transmitted parts of the Green’s function.

The DDA problem analogous to Equations (4) and (8) is then:

(14) $$\sum_{k=1}^{N}\left[{\overset{⃡}{G}}_{jk}^{\text{FS}}+{\overset{⃡}{G}}_{jk}^{1\text{D,ref}}\right]p_{k}=-\frac{\varepsilon_{0}c^{2}}{\omega^{2}}\left[E_{\text{inc},j}+E_{\text{inc}-\text{ref},j}\right].$$

Once the *N* dipole moments are found by solving the DDA problem, the scattered field at an arbitrary position **r** can be computed with Equation (6) using the 1D cylindrical Green’s functions in place of ${\overset{⃡}{G}}^{\text{ref}}$ and ${\overset{⃡}{G}}^{\text{tra}}$ .

The advantage of this 1D method is that it is very computationally efficient; the disadvantage is the instability of integration in the Green’s functions. Sommerfeld integrals with singular points are still involved and oscillation challenges still exist. Some of these singularities, e.g., at *ρ* = 0 are analytically removable using L’Hôpital’s rule (Appendix C). Two other singularities occur when the terms in the denominator of Equation (C3) equal zero: *k*_1*z*_ = 0, *k*_2*z*_ = 0 (Appendix C), which are *k*_*ρ*_ = *k*_1_, *k*_*ρ*_ = *k*_2_. We use the Gauss–Kronrod (GK) quadrature routine [40] to handle these singularities and evaluate ${\overset{⃡}{G}}^{1\text{D,ref}}$ with the assumption that loss in the background and substrate are negligible such that *k*_1_ and *k*_2_ are treated as real, which is typical for CMOS-compatible metasurfaces. The GK routine is an adaptive method for numerical integration, and it can automatically select the integration sampling points and choose proper (high or low) order quadrature rules to evaluate rapidly or slowly varying functions. The other advantage of the GK routine is that the end points of the integration range are unused. To take advantage of this, we split the interval of integration over *k*_*ρ*_ such that singularities are located at the boundaries of the three intervals: (0, *k*_1_), (*k*_1_, *k*_2_), and (*k*_2_, *k*_max_). Adding the results from these three intervals yields the full integral.

**Figure 3:**
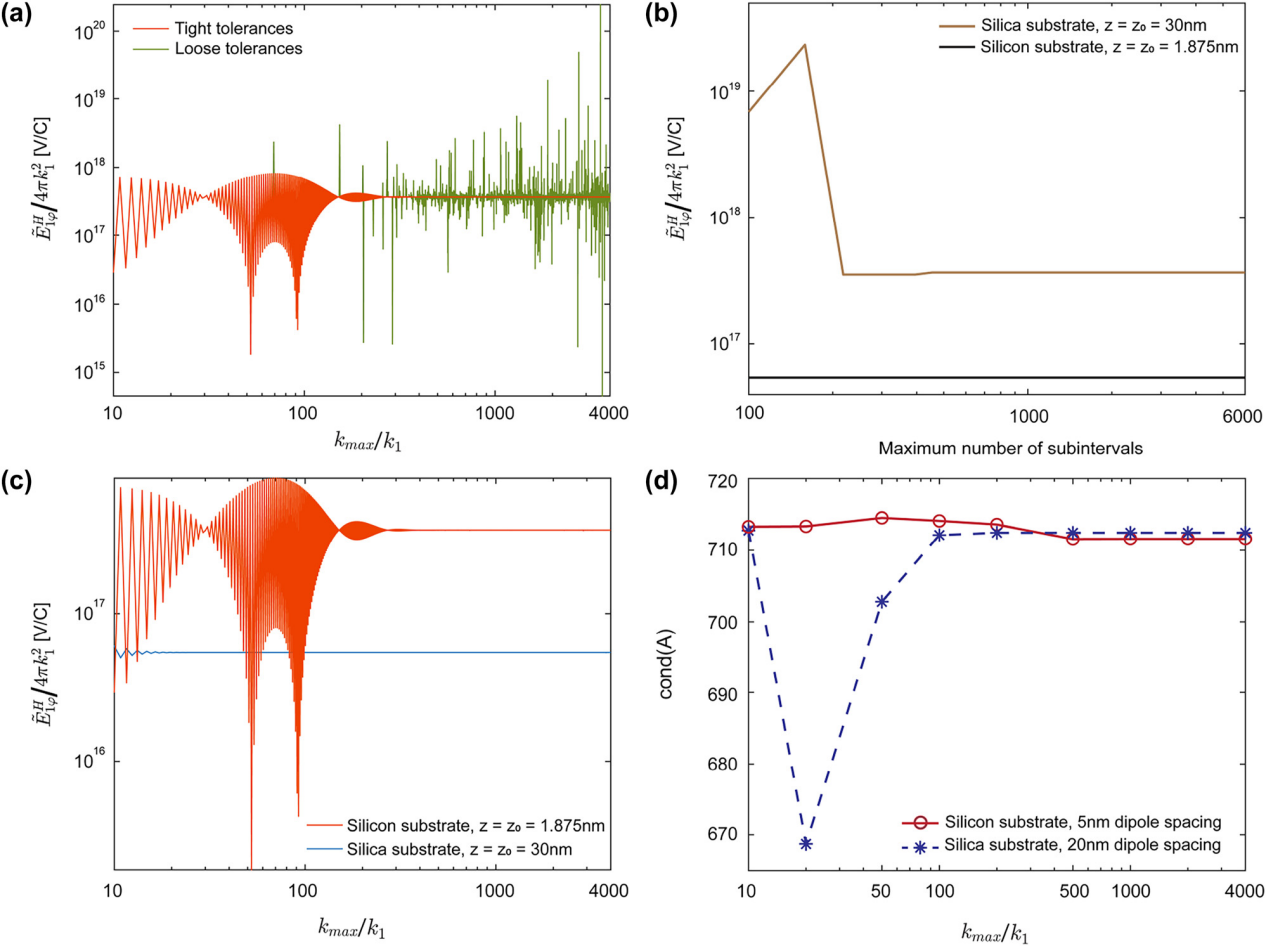
Convergence of numerical computation of the infinite 1D Green’s function integrals. Unless otherwise noted, the parameters for each panel are *ρ* = 2 μm, *z* = *z*_0_ = 1.8750 nm, *ɛ*_*b*_ = 1, *λ* = 1064 nm, and *ɛ*_sub_ = 3.5548^2^ = 12.6366. (a) Azimuthal component of a scaled electric field above the substrate resulting from a horizontally polarized dipole as a function of different integral truncation values *k*_max_ for two different sets of error tolerances. Loose tolerances correspond a relative tolerance of 10^−3^ and an absolute tolerance of 10^−3^ V/C, specified as options in quadgk. Tight tolerances correspond to a relative tolerance of 10^−7^ and an absolute tolerance of 10^−7^ V/C. (b) Scaled electric field as a function of the maximum number of subintervals used in quadgk for interval (*k*_2_, *k*_max_). The brown line corresponds to the default parameters, while the black line corresponds to *ɛ*_sub_ = 1.4496^2^ = 2.1013, *z* = *z*_0_ = 30 nm. (c) Convergence in electric field calculation for different dipole spacings and substrate materials. The orange line corresponds to the default parameters, while the blue line corresponds to *ɛ*_sub_ = 1.4496^2^ = 2.1013, *z* = *z*_0_ = 30 nm. (d) Condition number of matrix *A* for gold elliptic nanocylinders with different choices of *k*_max_ and different dipole spacings and substrate materials. The blue line corresponds to the system geometry illustrated in Section 2.1 with *a* = 300 nm, *b* = 90 nm, and *h* = 60 nm on a silica substrate with discretizing factor *D* = 3. The red line corresponds to *a* = 75 nm, *b* = 22.5 nm, and *h* = 15 nm on a silicon substrate with discretizing factor *D* = 3.

We use the built-in Matlab function quadgk to perform the GK routine. Several parameters must be considered for accurate and efficient computation: absolute and relative error, maximum number of subintervals, and the numerical truncation of *k*_*ρ*_ at *k*_max_ instead of the analytical integration to infinity. Figure 3(a) shows how ${\tilde{E}}_{1\phi }^{H}$ varies with different choices of *k*_max_ up to 4000*k*_1_ for two different sets of error tolerances. The spikes in the loose tolerance case show that the computation does not fully converge, whereas for the tight tolerance case, a smooth and converged result is found for any *k*_max_ > 600*k*_1_. The function quadgk automatically breaks the initial set of 3 intervals into many subintervals following the GK algorithm; however, the maximum number of subintervals must be defined by the user. Figure 3(b) shows how ${\tilde{E}}_{1\phi }^{H}$ changes as a function of the maximum number of subintervals used in quadgk. In general, more than 1000 subintervals are necessary.

Based on the results of Figure 3 panels (a) and (b), we fix the relative tolerance at 10^−7^ and absolute tolerance at 10^−7^ V/C and maximum number of subintervals at 6000 and investigate the convergence properties for different dipole locations and substrate materials (Figure 3(c)). Convergence is generally slower for smaller dipole spacings and higher substrate refractive indices. To further ensure all values in the coupling matrix $A=\alpha_{k}\varepsilon_{b}\left({\overset{⃡}{G}}_{jk}^{\text{FS}}+{\overset{⃡}{G}}_{jk}^{1\text{D,ref}}\right)$ have converged, we use the Matlab built-in function cond to compute the condition number of *A* under different cut-off *k*_max_ values until it converges, as illustrated in Figure 3(d), and we found converged results for *k*_max_ > 1000*k*_1_ in all cases.

Now, we can accurately and efficiently calculate ${\overset{⃡}{G}}_{jk}^{\text{FS}}+{\overset{⃡}{G}}_{jk}^{1\text{D,ref}}$ for any given *j*, *k*. The 9 terms in the free-space Green’s functions defined in Equation (3) remain unchanged when the dipole location **r**_0_ and observation point **r** are interchanged, so ${\overset{⃡}{G}}_{jk}^{\text{FS}}={\overset{⃡}{G}}_{kj}^{\text{FS}}$ . However, some terms in the reflected cylindrical Green’s functions change sign when **r**_0_ and **r** are interchanged. When this happens, *ρ* remains unchanged, *φ* → *φ* ± *π*, and *z* and *z*_0_ should be swapped. A close inspection of the reflected part of all Green’s functions (Equation (C1)) reveals that *φ* only appears in sin and cos functions, resulting in a sign change for these functions, while interchanging *z* and *z*_0_ does not result in any changes to the Green’s functions. Applying a sign change for each trigonometric function of *φ* in Equation (13) results in the symmetry relationship:

(15) $$\begin{array}{cc} if\;{\overset{⃡}{G}}_{jk}^{1\text{D,ref}} & =\left[\begin{array}{ccc} G_{11} & G_{12} & G_{13}\\ G_{21} & G_{22} & G_{23}\\ G_{31} & G_{32} & G_{33} \end{array}\right],\\ \;then\;{\overset{⃡}{G}}_{kj}^{1\text{D,ref}} & =\left[\begin{array}{ccc} G_{11} & G_{12} & -G_{13}\\ G_{21} & G_{22} & -G_{23}\\ -G_{31} & -G_{32} & G_{33} \end{array}\right]. \end{array}$$

Because the Green’s functions ${\overset{⃡}{G}}_{jk}^{\text{FS}}$ and ${\overset{⃡}{G}}_{kj}^{\text{FS}}$ are simply related to each other in both the free space and substrate cases, only the upper triangular matrix terms (*k* ≥ *j*) require time-consuming computation.

## 3 Results and discussion

### 3.1 Computational time

For metasurface design applications, both accuracy and computational time are important. With unlimited computational resources, the methods in Sections 2.2, 2.3, 2.4, and 2.5 would produce the same results. In reality, the performance varies significantly.

To compute the electromagnetic field scattered by a nanostructure, the DDA codes require time to execute several key steps: *t*_1_ to compute the incident field at all dipoles, *t*_2_ to build up the 3*N* × 3*N* Green’s function coupling matrix, *t*_3_ to solve the matrix problem for the unknown dipole moments, and *t*_4_ to compute the scattered field from all dipoles. We denote the individual computational times related to these steps as *s*_1_ to compute the incident field at a single dipole location, *s*_2_ to compute a single term in the coupling matrix by parallel computing, *s*_3_ to solve for all of the unknown dipole moments, and *s*_4_ to compute the field scattered by a single dipole over a monitor sampling area *q*_*x*_ × *q*_*y*_ = 102 × 108 points by parallel computing. Note that *t*_3_ = *s*_3_ as solving for the dipole moments is a single step.

To compare the computational times, we use the system geometry illustrated in Section 2.1 with a gold elliptic cylinder (*a* = 300 nm, *b* = 90 nm, *h* = 60 nm) on a silica substrate with discretizing factor *D* = 2 over a monitor size of 2 μm × 2 μm. For the 2D Cartesian and 1D cylindrical Green’s function methods, there are *N*_ns_ = 200 nanostructure dipoles in the system, and the monitor is located at *z* = −200 nm. For the substrate discretization method, there are an additional *N*_sub_ = 12,005 substrate dipoles, and monitor is located at *z* = 200 nm to avoid computing the fields inside the substrate dipoles. The results are shown in Table 1.

**Table 1:** Discrete dipole approximation computational times. Total time for each step (*t*) as well as individual element time (*s*) is provided. See the main text for the precise definitions of each of these steps. The simulation times correspond to specific single runs on our server, and thus our choice of reporting results to three significant figures is not intended to indicate any measure of uncertainty.

	Substrate discretization	2D Green’s function	1D Green’s function
*N*	12,205	200	200
*t*_1_[*s*]	1.15	5.96 × 10^−2^	5.86 × 10^−2^
*t*_2_[*s*]	389	1.69 × 10^4^	31.4
*t*_3_[*s*]	377	1.91 × 10^−2^	1.66 × 10^−2^
*t*_4_[*s*]	41.1	1.62 × 10^4^	22.2
*t* ^total^[*s*]	807	3.32 × 10^4^	53.6
*s*_1_[*s*]	9.43 × 10^−5^	2.98 × 10^−4^	2.93 × 10^−4^
*s*_2_[*s*]	8.70 × 10^−7^	2.34 × 10^−2^	3.12 × 10^−4^
*s*_3_[*s*]	377	1.92 × 10^−2^	1.66 × 10^−2^
*s*_4_[*s*]	3.37 × 10^−3^	81.1	0.111
Pattern error [%]	44.7	4.58	4.21
Power error [%]	3.04 × 10^3^	22.4	28.7

These simulations are executed on our lab’s server with specs: dual Intel Xeon CPU E5-2660 v3 @2.6 GHz processors, each with 10 real cores and 10 virtual cores that combine to provide 40 logical processors altogether, and 256 GB of 2133 MT/s RDIMM memory. We are able to use the parallel computing toolbox of Matlab to compute steps 2 and 4 described above because the 3 × 3 Green’s functions are independent of each other, as are the scattered fields from different dipoles with known dipole moments. Matlab is able to use 20 workers on our server, which is equivalent to ∼50 % CPU.

For all of our computational approaches, the time required to calculate the incident field at all dipole locations is small compared to the total time and, therefore, *t*_1_ can be neglected. The symmetry and antisymmetry between ${\overset{⃡}{G}}_{jk}$ and ${\overset{⃡}{G}}_{kj}$ discussed in the previous section reduce the computational scale of the coupling matrix from *fN* ^2^ to $\frac{f}{2}\left(N^{2}+N\right)$ , where *f* is the number of independent terms in each 3 × 3 Green’s function, which improves the computational efficiency for step *t*_2_.

For the substrate discretization method, the substrate is typically very large compared to the nanostructure, effectively extending to infinity. Therefore, a large *N*_sub_ is required for accurate results with minimal diffraction artifacts from truncation. We find that balancing the demand for computational resources against desired accuracy typically results in *N*_sub_ > 10^5^, while the nanostructure itself only requires *N*_ns_ ∼ 10^3^ or fewer due to its small, wavelength-scale size. So the number of substrate dipoles dominates the computational time. Furthermore, the calculation of the field at the monitor location is computationally similar to the calculation of the individual elements of the coupling matrix, so *s*_4,sd_ ≈ 6*s*_2,sd_ *q*_*x*_ *q*_*y*_, where the factor of 6 is the number of independent terms in the Green’s function. Combining all steps and applying symmetry and antisymmetry between ${\overset{⃡}{G}}_{jk}$ and ${\overset{⃡}{G}}_{kj}$ , the substrate discretization method requires a total time of

(16) $$\begin{array}{ll} t_{\text{sd}}^{\text{total}} & =t_{1,sd}+t_{2,sd}+t_{3,sd}+t_{4,sd}\\ & =\left(N_{\text{sub}}+N_{\text{ns}}\right)s_{1,sd}+3\left({\left(N_{\text{sub}}+N_{\text{ns}}\right)}^{2}\right.\\ & \left.\;+\left(N_{\text{sub}}+N_{\text{ns}}\right)\right)s_{2,sd}+s_{3,sd}+\left(N_{\text{sub}}+N_{\text{ns}}\right)s_{4,sd}\\ & \approx N_{\text{sub}}s_{1,sd}+3\left(N_{\text{sub}}^{2}+2N_{\text{sub}}q_{x}q_{y}\right)s_{2,sd}+s_{3,sd} \end{array}$$

For the 2D Cartesian Green’s function method, there are no substrate dipoles because substrate reflections are incorporated into the Green’s function itself, *N* = *N*_ns_. Again, *s*_4,2D_ ≈ 6*s*_2,2D_ *q*_*x*_ *q*_*y*_. The computational time is

(17) $$t_{2\text{D}}^{\text{total}}\approx N_{\text{ns}}s_{1,2D}+3\left(N_{\text{ns}}^{2}+2N_{\text{ns}}q_{x}q_{y}\right)s_{2,2D}+s_{3,2D}.$$

Compared to the substrate discretization method, the biggest advantage is that *N* is several orders of magnitude smaller. However, the value of *s*_2_ increases significantly because of the 2D Green’s function Sommerfeld integrals involved in Equation(7). On the other hand, *s*_3_ is significantly reduced compared to the substrate discretization method due to the smaller *N*.

For the 1D cylindrical Green’s function method, the computational time is similarly, $t_{1\text{D}}^{\text{total}}=N_{\text{ns}}s_{1,1D}+\frac{5}{2}\left(N_{\text{ns}}^{2}+N_{\text{ns}}\right)s_{2,1D}+s_{3,1D}+N_{\text{ns}}s_{4,1D}$ . Note that this Green’s function has only 5 independent terms, corresponding to ${\tilde{E}}_{n\rho }^{H}$ , ${\tilde{E}}_{n\varphi }^{H}$ , ${\tilde{E}}_{n\rho }^{V}$ , ${\tilde{E}}_{nz}^{H}$ , and ${\tilde{E}}_{nz}^{V}$ in Equation (13). In calculating the *N*_ns_ *s*_1,1D_ and $\frac{5}{2}\left(N_{\text{ns}}^{2}+N_{\text{ns}}\right)s_{2,1D}$ terms, the 1D Green’s function performs much better due to the reduced dimensionality of the Sommerfeld integral, as discussed in Section 2.5. In calculating the electromagnetic field across a monitor with sampling points *q*_*x*_ × *q*_*y*_, the computational time is significantly reduced from *s*_4,2D_ ≈ 5*s*_2,2D_ *q*_*x*_ *q*_*y*_ for the 2D method to *s*_4,1D_ ≈ 5*s*_2,1D_ *q*_max_, where $q_{\max}=\sqrt{{\left(q_{x}/2\right)}^{2}+{\left(q_{y}/2\right)}^{2}}$ , as illustrated in Figure 2. From panel (a), one would naively assume that 5*s*_2,1D_ *q*_*x*_ *q*_*y*_ Green’s function evaluations are still required, as was the case for the 2D method, However, for the 1D method, the time-consuming Sommerfeld integrals are independent of *φ*, as seen in Equations (9) and (10), even though the Green’s function is in general dependent on *φ* for horizontal dipoles. This means that the slow Sommerfeld integration need only be performed along one radial line, as illustrated in Figure 2(b), rather than for each pixel over the whole monitor area. To compute the Green’s function across the whole area, the vertical dipole results for the radial line can be used, while for horizontal dipoles, the Sommerfeld integrals only need to be multiplied by trigonometric functions of *φ*. This reduces the overall time complexity to *N*_ns_ *s*_4,1D_ ≈ 5*t*_2,1D_ *q*_max_. The total computational time is then,

(18) $$t_{1\text{D}}^{\text{total}}\approx N_{\text{ns}}s_{1,1D}+\frac{5}{2}\left(N_{\text{ns}}^{2}+2N_{\text{ns}}q_{\max}\right)s_{2,1D}+s_{3,1D}.$$

### 3.2 Dipole modeling effects on the DDA-with-substrate methods

**Figure 4:**
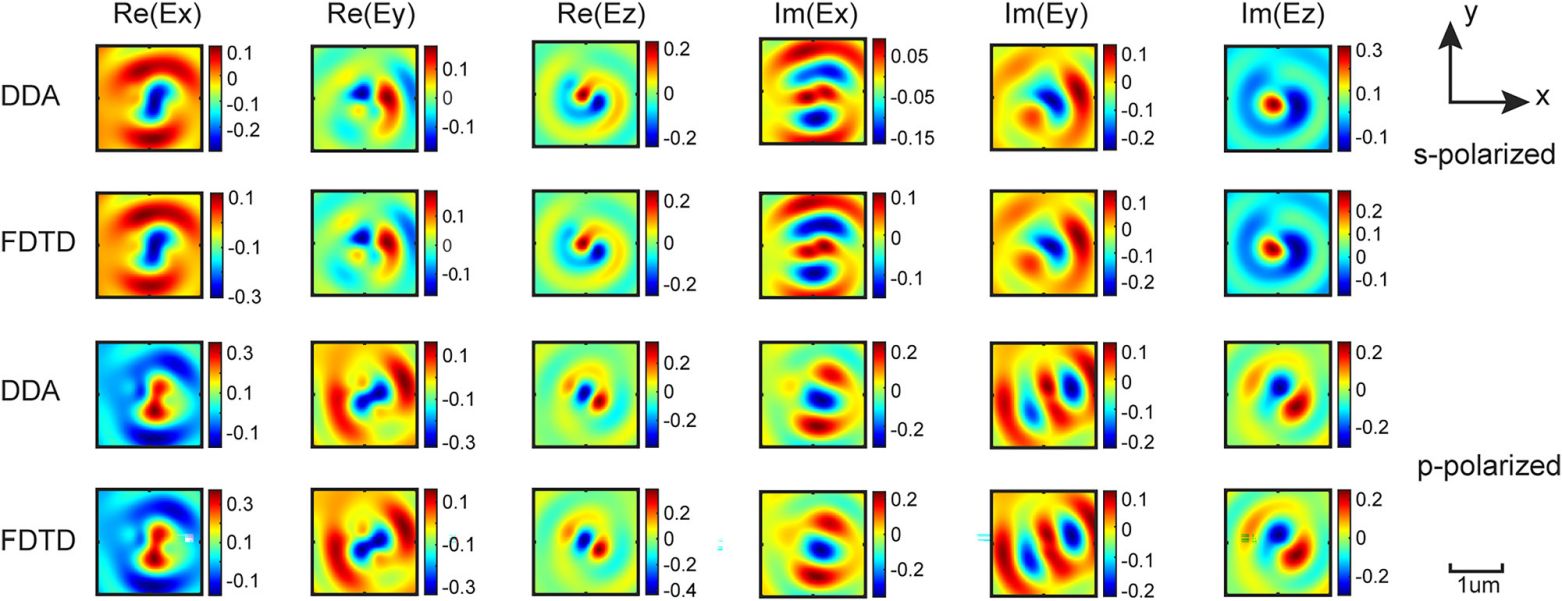
Converged 1D cylindrical Green’s function DDA results validated against FDTD. The real and imaginary parts of electric field computed by the 1D cylindrical Green’s function DDA and FDTD methods for the larger gold elliptic cylinder (*a* = 300 nm, *b* = 90 nm, *h* = 60 nm) at a discretizing factor *D* = 3.

We compare the real and imaginary parts of the electric field over surface *S* (see Figure 1) computed by our 1D Green’s function method to those from our gold standard FDTD simulations, as shown in Figure 4. Out of all the DDA methods, we concentrate on the 1D cylindrical Green’s function method, because it possesses the best computational performance as discussed above. The electric fields computed by the two methods are almost identical for all six electric field components. Considering that Matlab is only able to use physical cores and not virtual cores, only up to 50 % of server processors are utilized. For a fair comparison, we manually change the resource settings in Lumerical to ensure that the same number of processors are used in multithreading. The FDTD simulations take 1823 s, while the 1D Green’s function method only takes 293 s to converge.

To quantitatively compare the field difference between our 1D Green’s function method and the FDTD method, we introduce two parameters called pattern error and power error:

(19) $$\begin{array}{ll} \text{pattern } & \text{error}\\ & \;=\frac{1}{6}\underset{i=x,y,z}{\sum }\underset{j=R,I}{\sum }\left|1-\frac{\underset{S}{\int }|E_{\text{DDA},i}^{j}\;E_{\text{FDTD},i}^{j}|dS}{\sqrt{\underset{S}{\int }|E_{\text{DDA},i}^{j}{|}^{2}dS}\cdot \sqrt{\underset{S}{\int }|E_{\text{FDTD},i}^{j}{|}^{2}dS}}\right| \end{array}$$

(20) $$\text{power error}=\frac{1}{3}\underset{i=x,y,z}{\sum }\left|1-\frac{\underset{S}{\int }|E_{\text{DDA},i}{|}^{2}dS}{\underset{S}{\int }|E_{\text{FDTD},i}{|}^{2}dS}\right|$$

The pattern error pertains to the calculation of correlation coefficients between the real and imaginary components of the *x*, *y*, and *z* components of the electric field over surface *S* between the 1D cylindrical Green’s function and FDTD methods. A correlation coefficient of 1 indicates a perfect match between these two methods, discounting any potential total power discrepancy, while 0 signifies no match at all. The pattern error quantifies the average deviation of each component from a 100 % similarity between these two methods. The power error captures the average power percentage differences of the electric field. Significantly, the pattern error holds greater importance as it is predominantly shaped by two key factors: the relative Green’s function values across *S* and the relative dipole moments of all particles. Conversely, the power error is primarily attributed to variations in polarizabilities computed from different dipole models, and its mitigation hinges on the pursuit of more precise dipole models. Furthermore, total power errors could be corrected by a simple constant multiplicative correction factor, whereas pattern errors cannot be simply corrected.

A prerequisite of accurate DDA simulations is a precise dipole model. Point dipoles, by definition, have infinitesimal volumes and, therefore, are theoretically approximated models. As mentioned in Section 2.3, small particles can be approximated as dipoles, and their dipole moments can by computed by Equation (1). The most common dipole model used to compute the polarizabilities *α* is the Clausius–Mossotti (CM) relation [43]:

(21) $$\alpha_{\text{CM}}=3\varepsilon_{0}V\frac{\varepsilon -\varepsilon_{b}}{\varepsilon +2\varepsilon_{b}}$$

where *ɛ* is the complex permittivity of the particle, *V* = *d* ^3^ is the volume of cubic voxels, and *d* is the dipole spacing. The reason that we use cubic voxels is so that the discretized dipoles are close-packed and occupy the full volume of the nanostructure. For improved accuracy for larger *d*, one can account for the back-action of the scattered field on the dipole using the radiation reaction correction (RR) [44]:

(22) $$\alpha_{\text{RR}}=\alpha_{\text{CM}}{\left(1-\frac{ik^{3}}{6\pi \varepsilon_{0}}\alpha_{\text{CM}}\right)}^{-1}.$$

The digitized Green’s function (DGF) dipole model includes corrections on the order of $O\left({\left(kd\right)}^{2}\right)$ , and it was proposed by Goedecke and O’Brien [45] based on cubical subvolume dipole:

(23) $$\alpha_{\text{DGF}}=\alpha_{\text{CM}}{\left[1-\frac{\alpha_{\text{CM}}}{4\pi \varepsilon_{0}d^{3}}\left(1.611992{\left(kd\right)}^{2}+\frac{2i}{3}{\left(kd\right)}^{3}\right)\right]}^{-1}$$

To accurately reproduce wave propagation and the dispersion relation in an infinite medium, the lattice dispersion relation (LDR) dipole model was proposed by Draine and Goodman [46] as:

(24) $$\begin{array}{ll} \alpha_{\text{LDR}} & =\alpha_{\text{CM}}\left\{1-\frac{\alpha_{\text{CM}}}{4\pi \varepsilon_{0}d^{3}}\left[\left(1.8915316-0.1648469\frac{\varepsilon }{\varepsilon_{b}}\right.\right.\right.\\ & \;{\left.\left.\left.+1.770004\frac{\varepsilon }{\varepsilon_{b}}S\right){\left(kd\right)}^{2}+\frac{2i}{3}{\left(kd\right)}^{3}\right]\right\}}^{-1}, \end{array}$$

where $S=\sum_{i}{\left(a_{i}e_{i}\right)}^{2},i=x,y,z$ , and *a*_*i*_ stands for the components of the unit vector for the direction of propagation and *e*_*i*_ stands for the components of the unit vector for the polarization of incident field.

**Figure 5:**
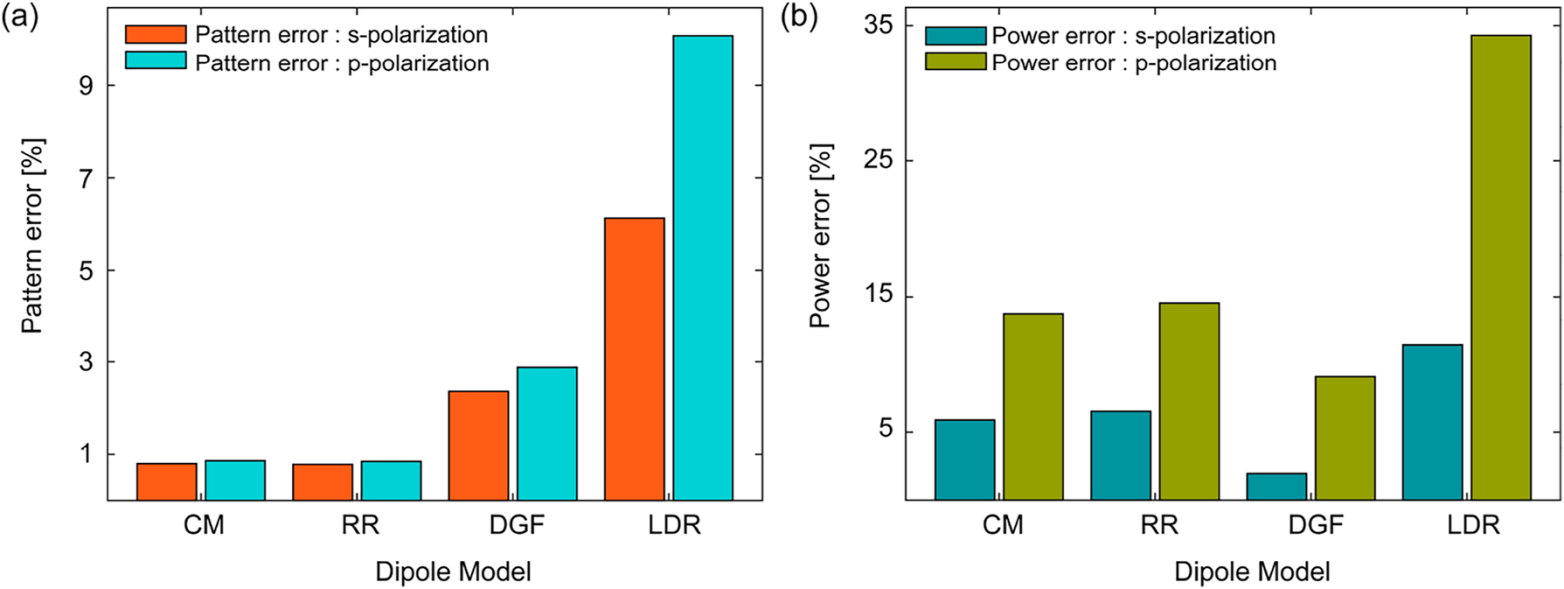
Effect of different dipole models. (a) Effect on pattern error. (b) Effect on power error.

We evaluated these four dipole models using the large gold elliptical cylinder (*a* = 300 nm, *b* = 90 nm, *h* = 60 nm) on a silica substrate with *D* = 3. The pattern and power errors shown in Figure 5 are calculated over a 2 μm × 2 μm monitor located at *z* = −200 nm. FDTD simulation results again serve as our gold-standard. Among these four dipole models, the CM and RR models provided less than 1 % pattern error for both polarizations. However, the power errors are slightly larger, ranging from 5 % to 15 %. We consider pattern errors as a more fundamental error, since power errors can be corrected by simply multiplying a constant factor. The DGF dipole model gives the best balance between pattern and power error, with pattern errors around 3 % for both polarizations, and power errors around 7 %. The cubical subvolume dipole assumption makes the DGF model have the best power errors among all four dipole models. The LDR dipole model gives the worst performance for both pattern and power errors, and therefore we do not recommend it for metasurface simulations.

### 3.3 Materials and shape effects on the DDA-with-substrate methods

**Figure 6:**
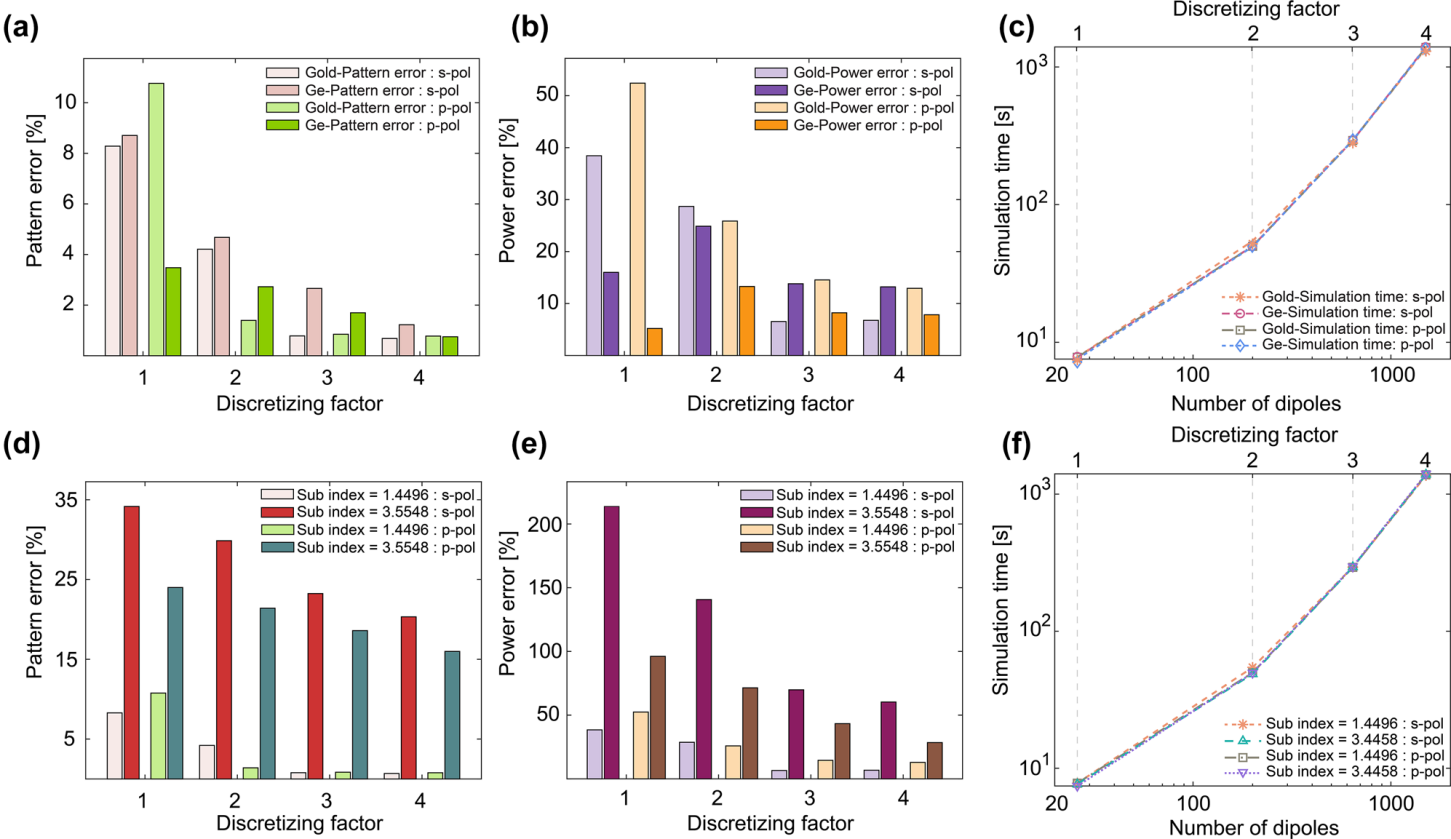
Comparison between the 1D cylindrical Green’s function method and the FDTD method. A large elliptic cylinder (*a* = 300 nm, *b* = 90 nm, *h* = 60) is simulated. (a)–(c) Results for different nanoparticle materials on a silica substrate illuminated with different polarizations. (d)–(f) Results for a gold nanoparticle on different substrate materials, illuminated with different polarizations.

We proceeded to assess the impact of different materials on the performance of the DDA-with-substrate methods. Figure 6 panel (a) shows how pattern errors change as a function of discretizing factor *D*. We tested two different particle materials: gold and germanium under both *s*- and *p*-polarization because metallic and high-index dielectric blocks are widely applied in metasurface applications. In all cases, the pattern errors reduce rapidly and reach our chosen convergence criteria: pattern error ≤5% , which corresponds to an average 95 % pattern similarity among all real and imaginary parts of the *x*, *y*, and *z* components of the electric field over surface *S*, when *D* ≥ 3. We also found that the overall pattern errors for germanium nanoparticles are slightly larger than for gold nanoparticles but are still less than 1 %, which indicates that the 1D Green’s function method can be generally applied to a variety of particle materials, including metals and high-index dielectrics. Similarly, in panel (b), the power errors also drop quickly for both materials and polarizations when the structure is more finely discretized. Here, there is a noticeable difference between gold and germanium nanoparticles: when *D* = 3, 4, for gold nanoparticles, the power errors of *s*-polarization are smaller than *p*-polarization, and on the contrary, for germanium nanoparticles, the power errors of *s*-polarization are larger than *p*-polarization. Panel (c) of Figure 6 shows how computational time scales with dipole number and discretizing factor for four cases, and it is apparent that the computational time is independent of polarization state and particle material, and overall scales with number of dipoles somewhere between *N* and *N* ^2^, but closer to *N*. A simulation with a few hundred dipoles can be performed on the order of minutes by using 1D Green’s function method.

Some integrated optics applications that require CMOS-compatible fabrication techniques and reflective metasurfaces typically use silicon as the substrate, so we also investigate how the substrate refractive index affects the simulation accuracy. We examine gold nanoparticles, keeping the other parameters the same. Figure 6 panels (d) and (e) compare the silicon and silica substrate. The pattern errors and power errors are significantly greater for the silicon substrate under both polarizations. Even at large discretizing factor *D* = 4, the pattern errors and power errors are approximately 20 % and 45 %, respectively.

**Figure 7:**
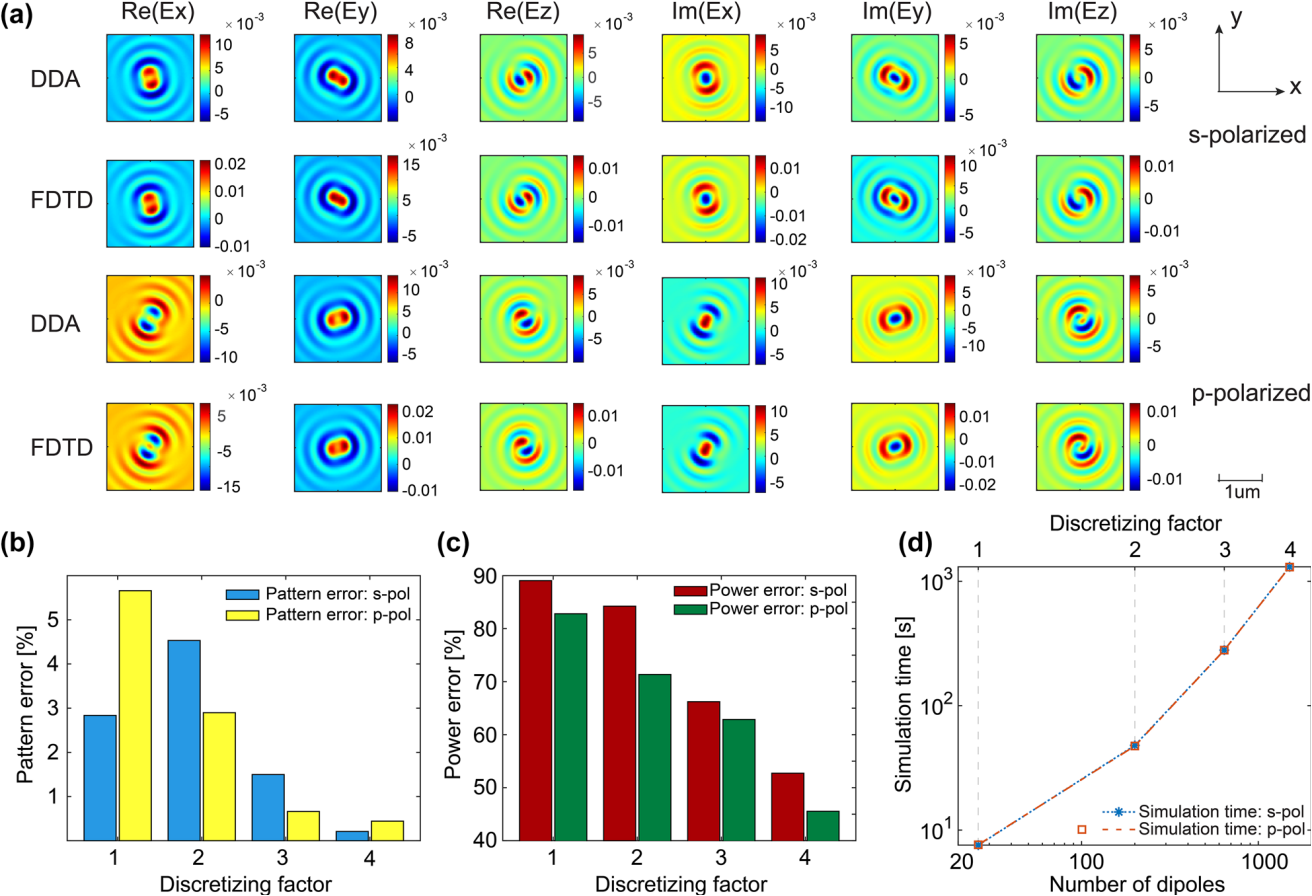
DDA-with-substrate simulation results for the smaller gold elliptic cylinder (*a* = 75 nm, *b* = 22.5 nm, *h* = 15 nm). (a) The real and imaginary parts of electric field computed by the 1D cylindrical Green’s function DDA-with-substrate method validated against FDTD calculations for the high refractive index substrate (silicon) with a discretizing factor *D* = 3. The patterns between the two methods are similar, while the power differences are greater. (b) Pattern error between the DDA and FDTD simulations. (c) Power error between the two simulations. (d) Computational time scaling of the DDA simulations.

Our server memory limits our ability to test cases with *D* > 4. So in order to test smaller dipole spacings, we shrink the dimensions of the elliptic cylinder to *a* = 75 nm, *b* = 22.5 nm, and *h* = 15 nm, which makes it quarter-scale compared to the original elliptic cylinder. Considering the definition of discretizing factor, *D* = 1, 2, 3, and 4 for small elliptic cylinders corresponds to the same dipole spacing as *D* = 4, 8, 12, and 16 in large elliptic nanocylinders. The recorded electric field over surface *S* in Figure 7 with a silicon substrate shows excellent pattern agreement and acceptable power agreement between the 1D Green’s function and the FDTD method. Panels (b) and (c) in Figure 7 show that the pattern errors and power errors change as a function of discretizing factor. Compared with the large elliptic cylinder, the pattern and power error reduce significantly for both polarizations. For pattern errors, they reach our convergence criteria when *D* = 2, and for power errors, we believe that they may be further reduced by using smaller dipole spacing. In actual applications, the power error is less important than the pattern error, as we can scale the electromagetic fields by multiplying a constant to correct power errors, rather than using smaller dipole spacing. Panel (d) of Figure 7 shows similar computational time and scaling as for the large elliptic cylinder in Figure 6.

We also investigated the impact of nanostructure shape dimensions on pattern and power errors by conducting two more tests on elliptical cylinders as depicted in Figure 1(a). The dimensions of these structures and results are presented in Table 2. For a fair comparison, we selected the same source wavelength and incidence angle as before, as well as the same monitor surface *S*. In this comparison, the elliptical cylinders are composed of germanium, and the substrates are silica. One of the elliptical cylinders is taller, with only a few dipoles touching the substrate, while the other is flatter, with many dipoles in contact with the substrate. Across all three elliptical cylinders with different dimensions, the pattern error remains largely consistent, whereas the power error increases as more dipoles come into contact with the substrate. At present, determining a correction factor for the overall power requires corresponding FDTD simulations.

**Table 2:** Accuracy of the 1D cylindrical Green’s function for elliptical cylinders of different dimensions. The pattern error is consistently low, while the power error increases when more dipoles are in close proximity to the substrate. In all cases, the dipole pitch is 20 nm.

	Standard elliptical cylinder	Flatter elliptical cylinder	Taller elliptical cylinder
Dimensions (*a*, *b*, *h*) [nm]	(300,90,60)	(200,100,40)	(50,50,100)
Short axis angle	*ϕ* = 150°	*ϕ* = 45°	*ϕ* = 0°
*N*	642	320	105
Pattern error [%]	2.66	1.08	0.064
Power error [%]	13.80	47.37	14.75

The above simulations and comparisons illustrate the main advantage of the 1D cylindrical Green’s function over techniques such as FDTD: the computational time scales with number of particles and monitor sampling points rather than domain size. This allows for monitors of arbitrary size and far field calculations without requiring a projection from the near field. As a result, this method can be used for many applications that cannot be simulated by FDTD, such as weak coupling of distant small particles and accurate scattering patterns over large simulation regions (e.g., in wafer inspection). Another advantage of the DDA approach over “black-box” machine learning techniques is that the dipole moment distribution throughout the structure can be used to provide physical understanding of what parts structure contribute the most to its optical properties.

There are a few limitations of our 1D cylindrical Green’s function method. Firstly, it is difficult to deal with nonlinear optical properties, specifically second harmonic generation from nanoparticles [38] or four-wave mixing in metasurfaces [39]. Equation (1) assumes a linear relationship between the polarization and local (driving) field. For nonlinear optical properties, higher order local fields like **E**_*j*_ ⊗**E**_*j*_ need to be incorporated into the dipole moment. Secondly, our approach currently models a domain in isolation, rather than a periodic array. However, thousands of dipoles can be simulated, and these dipoles need not be in the same metasurface particle, so modest arrays of elements could be simulated. Infinite periodic arrays can be simulated using a generalized DDA formulation, as outlined in Draine and Flatau’s 2008 work [47] or by applying the Bloch theorem to compute the effective polarizability of nanoparticles in a lattice, as discussed in the paper [48]. Furthermore, reference [48] provides analytical expressions for using the DDA method to compute the scattering matrix between layers in rigorous coupled-wave analysis (RCWA). However, it encounters challenges related to low convergence and accuracy near an interface. Our 1D cylindrical Green’s function method may serve as a valuable tool to address these challenges in the future.

## 4 Conclusions

We tested our DDA-with-substrate method for metallic and dielectric particle materials, both low and high refractive index substrate materials, different sizes of structures, and different polarization states of incident light. Our results are for a wavelength of 1064 nm, and while there is nothing special about this wavelength, extrapolation to other wavelengths would need to be verified. The DDA-with-substrate method using a 1D cylindrical Green’s function is a general, efficient, and accurate method for the scattered field calculation from structures on substrate and can be widely used in metasurface design applications. Easier systems, such as dielectric particles with low indices, can also be simulated with low error. Note that although the Green’s function approach generalizes to any kind of monitor, our 1D cylindrical Green’s function method is particularly efficient for monitor planes perpendicular to the *z*-axis (see discussion preceding Equation (18)). Compared with the FDTD method, the computational time of our DDA method is at least one order of magnitude faster with the same level of accuracy for the low refractive index substrate. For high refractive index substrates like silicon, our method achieves a 98 % similarity in the electric field pattern, however, with a relatively large power error that may be mitigated by applying a correction factor. All the codes are written in Matlab, and the metasurface design process can be implemented without purchasing FDTD or FEM software.

## Appendix A: Cartesian Green’s function

The equations in this appendix follow the derivation from reference [40]. The analytical solutions of a dipole near layered substrate can be expressed as 2D integrations in (*k*_*x*_, *k*_*y*_) space (Equation (7)). The two parts of the integrand in Equation (7) are defined as,

(A1)
$$\begin{array}{cc} {\overset{⃡}{M}}_{\text{ref}}^{s} & =\frac{r^{s}\left(k_{x},k_{y}\right)}{k_{1z}\left(k_{x}^{2}+k_{y}^{2}\right)}\left[\begin{array}{ccc} k_{y}^{2} & -k_{x}k_{y} & 0\\ -k_{x}k_{y} & k_{x}^{2} & 0\\ 0 & 0 & 0 \end{array}\right],\\ {\vec{M}}_{\text{ref}}^{p} & =\frac{-r^{p}\left(k_{x},k_{y}\right)}{k_{1}^{2}\left(k_{x}^{2}+k_{y}^{2}\right)}\\ & \;\times \left[\begin{array}{ccc} k_{x}^{2}k_{1z} & k_{x}k_{y}k_{1z} & k_{x}\left(k_{x}^{2}+k_{y}^{2}\right)\\ k_{x}k_{y}k_{1z} & k_{y}^{2}k_{1z} & k_{y}\left(k_{x}^{2}+k_{y}^{2}\right)\\ -k_{x}\left(k_{x}^{2}+k_{y}^{2}\right) & -k_{y}\left(k_{x}^{2}+k_{y}^{2}\right) & -{\left(k_{x}^{2}+k_{y}^{2}\right)}^{2}/k_{1z} \end{array}\right]. \end{array}$$

The transmitted Green’s function in layer 2 of Figure 1 is:

(A2)
$$\begin{array}{ll} {\overset{⃡}{G}}_{\text{tra}}\left(r,r_{0}\right) & =\frac{i}{8\pi^{2}}\overset{\infty }{\underset{-\infty }{\iint }}\left[{\overset{⃡}{M}}_{\text{tra}}^{s}\left(k_{x},k_{y}\right)+{\overset{⃡}{M}}_{\text{tra}}^{p}\left(k_{x},k_{y}\right)\right]\\ & \;\times e^{\text{i}\left[\left.k_{x}\left(x-x_{0}\right)+k_{y}\left(y-y_{0}\right)+k_{1z}z_{0}-k_{2z}z\right)\right]}\;dk_{x}\;dk_{y}. \end{array}$$

Similarly, ${\overset{⃡}{M}}_{\text{tra}}^{s}$ and ${\overset{⃡}{M}}_{\text{tra}}^{p}$ are defined as:

(A3)
$$\begin{array}{cc} {\overset{⃡}{M}}_{\text{tra}}^{s} & =\frac{t^{s}\left(k_{x},k_{y}\right)}{k_{1z}\left(k_{x}^{2}+k_{y}^{2}\right)}\left[\begin{array}{ccc} k_{y}^{2} & -k_{x}k_{y} & 0\\ -k_{x}k_{y} & k_{x}^{2} & 0\\ 0 & 0 & 0 \end{array}\right],\\ {\overset{⃡}{M}}_{\text{tra}}^{p} & =\frac{t^{p}\left(k_{x},k_{y}\right)}{k_{1}k_{2}\left(k_{x}^{2}+k_{y}^{2}\right)}\\ & \;\times \left[\begin{array}{ccc} k_{x}^{2}k_{2z} & k_{x}k_{y}k_{2z} & k_{x}\left(k_{x}^{2}+k_{y}^{2}\right)k_{2z}/k_{1z}\\ k_{x}k_{y}k_{2z} & k_{y}^{2}k_{2z} & k_{y}\left(k_{x}^{2}+k_{y}^{2}\right)k_{2z}/k_{1z}\\ k_{x}\left(k_{x}^{2}+k_{y}^{2}\right) & k_{y}\left(k_{x}^{2}+k_{y}^{2}\right) & {\left(k_{x}^{2}+k_{y}^{2}\right)}^{2}/k_{1z} \end{array}\right]. \end{array}$$

The variables *r* ^*s*^(*k*_*x*_, *k*_*y*_), *r* ^*p*^(*k*_*x*_, *k*_*y*_), *t* ^*s*^(*k*_*x*_, *k*_*y*_), and *t* ^*p*^(*k*_*x*_, *k*_*y*_) in Equations (A1) and (A3) are the Fresnel coefficients and can be computed as:

(A4)
$$\begin{array}{cc} r^{s}\left(k_{x},k_{y}\right)= & \frac{\mu_{2}k_{1z}-\mu_{1}k_{2z}}{\mu_{2}k_{1z}+\mu_{1}k_{2z}},\\ r^{p}\left(k_{x},k_{y}\right)= & \frac{\varepsilon_{2}k_{1z}-\varepsilon_{1}k_{2z}}{\varepsilon_{2}k_{1z}+\varepsilon_{1}k_{2z}}.\\ t^{s}\left(k_{x},k_{y}\right)= & \frac{2\mu_{2}k_{1z}}{\mu_{2}k_{1z}+\mu_{1}k_{2z}},\\ t^{p}\left(k_{x},k_{y}\right)= & \frac{2\varepsilon_{2}k_{1z}}{\varepsilon_{2}k_{1z}+\varepsilon_{1}k_{2z}}\sqrt{\frac{\mu_{2}\varepsilon_{1}}{\mu_{1}\varepsilon_{2}}}. \end{array}$$

With an assumption of dielectric substrate materials, the singular points in ${\overset{⃡}{G}}^{\text{ref}}\left(r,r_{0}\right)$ and ${\overset{⃡}{G}}^{\text{tra}}\left(r,r_{0}\right)$ are located where the denominators of matrices ${\overset{⃡}{M}}_{\text{ref}}^{s}$ , ${\overset{⃡}{M}}_{\text{ref}}^{p}$ , ${\overset{⃡}{M}}_{\text{tra}}^{s}$ , and ${\overset{⃡}{M}}_{\text{tra}}^{p}$ equal 0. Therefore, the singular points in the 2D Green’s function methods are at: $k_{x}^{2}+k_{y}^{2}=0$ , $k_{x}^{2}+k_{y}^{2}=k_{1}^{2}$ , and $k_{x}^{2}+k_{y}^{2}=k_{2}^{2}$ .

## Appendix B: trapz for 2D Sommerfeld integration

Accurate integration using trapz() requires varying *k*_max_ and the number of sampling points in frequency space for different dipole heights *z*_0_. The values used in this paper are listed in Table B1.

**Table B1:** Parameters for calculating 2D Cartesian Green’s functions using the built-in Matlab function trapz.

For dipole heights	Parameters for ${\overset{⃡}{G}}_{\text{ref}}$		For dipole heights	Parameters for ${\overset{⃡}{G}}_{\text{tra}}$	
*z*_0_ in the range	*k*_max_	Sampling points	*z*_0_ in the range	*k*_max_	Sampling points
(0, 20] nm	250*k*_1_	3104 × 3106	(0, 10] nm	180*k*_1_	2104 × 2106
(20, 100] nm	80*k*_1_	1204 × 1206	(10, 100] nm	60*k*_1_	804 × 806
(100, 250] nm	30*k*_1_	804 × 806	(100, 250] nm	30*k*_1_	504 × 506
(250, 500] nm	10*k*_1_	504 × 506	(250, 500] nm	10*k*_1_	204 × 206
(500, 1000] nm	5*k*_1_	204 × 206	(500, 1000] nm	5*k*_1_	104 × 106
(1000, ∞) nm	3*k*_1_	104 × 106	(1000, ∞) nm	3*k*_1_	54 × 56

## Appendix C: Cylindrical Green’s function

The equations in this appendix are adapted from reference [40] from a 3-layer system to a 2-layer system. The analytical solutions of a dipole a near layered substrate can be expressed as 1D integrations in *k*_*ρ*_ space. The scattered fields in layer 1 of Figure 1, including those reflected off the substrate, are given by:

(C1)
$$\begin{array}{ll} E_{1\rho }^{V} & =p_{z}{\tilde{E}}_{1\rho }^{V}\\ & =\rho \left(z-z_{0}\right)\frac{p_{z}}{4\pi \varepsilon_{0}\varepsilon_{1}}\frac{e^{\text{i}k_{1}R}}{R^{3}}\left[\frac{3}{R^{2}}-\frac{3ik_{1}}{R}-k_{1}^{2}\right]\\ & \;-\frac{ip_{z}}{4\pi \varepsilon_{0}\varepsilon_{1}}\overset{\infty }{\underset{0}{\int }}J_{1}\left(k_{\rho }\rho \right)A_{1}k_{\rho }k_{1z}e^{\text{i}k_{1z}\left(z+z_{0}\right)}dk_{\rho },\\ E_{1\varphi }^{V} & =0,\\ E_{1z}^{V} & =p_{z}{\tilde{E}}_{1z}^{V}\\ & =\frac{p_{z}}{4\pi \varepsilon_{0}\varepsilon_{1}}\frac{e^{\text{i}k_{1}R}}{R}\left[\frac{3{\left(z-z_{0}\right)}^{2}}{R^{4}}-\frac{3ik_{1}{\left(z-z_{0}\right)}^{2}}{R^{3}}\right.\\ & \;\left.-\frac{1+k_{1}^{2}{\left(z-z_{0}\right)}^{2}}{R^{2}}+\frac{ik_{1}}{R}+k_{1}^{2}\right]\\ & \;+\frac{p_{z}}{4\pi \varepsilon_{0}\varepsilon_{1}}\overset{\infty }{\underset{0}{\int }}J_{0}\left(k_{\rho }\rho \right)A_{1}k_{\rho }^{2}e^{\text{i}k_{1z}\left(z+z_{0}\right)}dk_{\rho },\\ E_{1\rho }^{H} & =\cos\varphi \;p_{x}\;{\tilde{E}}_{1\rho }^{H}\\ & =\cos\varphi \frac{p_{x}}{4\pi \varepsilon_{0}\varepsilon_{1}}\frac{e^{\text{i}k_{1}R}}{R}\left\{\left[k_{1}^{2}+\frac{ik_{1}}{R}-\frac{1}{R^{2}}\right]\right.\\ & \left.\;+\frac{\rho^{2}}{R^{2}}\left[\frac{3}{R^{2}}-\frac{3ik_{1}}{R}-k_{1}^{2}\right]\right\}+\cos\varphi \frac{p_{x}}{4\pi \varepsilon_{0}\varepsilon_{1}}\\ & \;\times \overset{\infty }{\underset{0}{\int }}e^{\text{i}k_{1z}\left(z+z_{0}\right)}\left\{\frac{1}{\rho }J_{1}\left(k_{\rho }\rho \right)\left[k_{\rho }B_{1}-ik_{1z}C_{1}\right]\right.\\ & \left.\;-ik_{1z}J_{0}\left(k_{\rho }\rho \right)\left[ik_{1z}B_{1}-k_{\rho }C_{1}\right]\right\}dk_{\rho },\\ E_{1\varphi }^{H} & =\sin\varphi \;p_{x}\;{\tilde{E}}_{1\varphi }^{H}\\ & =\sin\varphi \frac{p_{x}}{4\pi \varepsilon_{0}\varepsilon_{1}}\frac{e^{\text{i}k_{1}R}}{R}\left[\frac{1}{R^{2}}-\frac{ik_{1}}{R}-k_{1}^{2}\right]\\ & \;+\sin\varphi \frac{p_{x}}{4\pi \varepsilon_{0}\varepsilon_{1}}\overset{\infty }{\underset{0}{\int }}e^{\text{i}k_{1z}\left(z+z_{0}\right)}\left\{\frac{1}{\rho }J_{1}\left(k_{\rho }\rho \right)\left[k_{\rho }B_{1}\right.\right.\\ & \left.\left.\;-ik_{1z}C_{1}\right]-k_{1}^{2}J_{0}\left(k_{\rho }\rho \right)B_{1}\right\}dk_{\rho },\\ E_{1z}^{H} & =\cos\varphi \;p_{x}\;{\tilde{E}}_{1z}^{H}\\ & =\cos\varphi \frac{p_{x}}{4\pi \varepsilon_{0}\varepsilon_{1}}\rho \left(z-z_{0}\right)\frac{e^{\text{i}k_{1}R}}{R^{3}}\left[\frac{3}{R^{2}}-\frac{3ik_{1}}{R}-k_{1}^{2}\right]\\ & \;-\cos\varphi \frac{p_{x}}{4\pi \varepsilon_{0}\varepsilon_{1}}\overset{\infty }{\underset{0}{\int }}e^{\text{i}k_{1z}\left(z+z_{0}\right)}\\ & \;\times k_{\rho }J_{1}\left(k_{\rho }\rho \right)\left[ik_{1z}B_{1}-k_{\rho }C_{1}\right]dk_{\rho }. \end{array}$$

where the superscript *H* represents a horizontal dipole and the superscript *V* represents for the vertical dipole. See below Equation (10) in the main text for parameter definitions. The electric field in layer 2, which is illustrated in Figure 1, can be expressed as:

(C2)
$$\begin{array}{ll} E_{2\rho }^{V} & =p_{z}{\tilde{E}}_{2\rho }^{V}\\ & =\frac{ip_{z}}{4\pi \varepsilon_{0}\varepsilon_{1}}\overset{\infty }{\underset{0}{\int }}J_{1}\left(k_{\rho }\rho \right)A_{2}k_{\rho }k_{2z}e^{\text{i}\left(k_{1z}z_{0}-k_{2z}z\right)}dk_{\rho },\\ E_{2\varphi }^{V} & =0,\\ E_{2z}^{V} & =p_{z}{\tilde{E}}_{2z}^{V}\\ & =\frac{p_{z}}{4\pi \varepsilon_{0}\varepsilon_{1}}\overset{\infty }{\underset{0}{\int }}J_{0}\left(k_{\rho }\rho \right)A_{2}k_{\rho }^{2}e^{\text{i}\left(k_{1z}z_{0}-k_{2z}z\right)}dk_{\rho },\\ E_{2\rho }^{H} & =\cos\varphi \;p_{x}\;{\tilde{E}}_{2\rho }^{H}\\ & =\cos\varphi \frac{p_{x}}{4\pi \varepsilon_{0}\varepsilon_{1}}\overset{\infty }{\underset{0}{\int }}e^{\text{i}\left(k_{1z}z_{0}-k_{2z}z\right)}\left\{\frac{1}{\rho }J_{1}\left(k_{\rho }\rho \right)\right.\\ & \;\times \left[k_{\rho }B_{2}+ik_{2z}C_{2}\right]-ik_{2z}J_{0}\left(k_{\rho }\rho \right)\\ & \;\times \left.\left[ik_{2z}B_{2}+k_{\rho }C_{2}\right]\right\}dk_{\rho },\\ E_{2\varphi }^{H} & =\sin\varphi \;p_{x}\;{\tilde{E}}_{2\varphi }^{H}\\ & =\sin\varphi \frac{p_{x}}{4\pi \varepsilon_{0}\varepsilon_{1}}\overset{\infty }{\underset{0}{\int }}e^{\text{i}\left(k_{1z}z_{0}-k_{2z}z\right)}\left\{\frac{1}{\rho }J_{1}\left(k_{\rho }\rho \right)\right.\\ & \;\left.\times \left[k_{\rho }B_{2}+ik_{2z}C_{2}\right]-k_{2}^{2}J_{0}\left(k_{\rho }\rho \right)B_{2}\right\}dk_{\rho },\\ E_{2z}^{H} & =\cos\varphi \;p_{x}\;{\tilde{E}}_{2z}^{H}\\ & =\cos\varphi \frac{p_{x}}{4\pi \varepsilon_{0}\varepsilon_{1}}\overset{\infty }{\underset{0}{\int }}e^{\text{i}\left(k_{1z}z_{0}-k_{2z}z\right)}k_{\rho }J_{1}\left(k_{\rho }\rho \right)\\ & \;\times \left[ik_{2z}B_{2}+k_{\rho }C_{2}\right]dk_{\rho }. \end{array}$$

The *A*, *B*, and *C* coefficients in Equations (C1) and (C2) are given by:

(C3)
$$\begin{array}{cc} A_{1}\left(k_{\rho }\right) & =i\frac{k_{\rho }f_{1}}{k_{1z}f_{2}},\\ A_{2}\left(k_{\rho }\right) & =i\frac{2\varepsilon_{1}k_{\rho }}{f_{2}},\\ B_{1}\left(k_{\rho }\right) & =i\frac{k_{\rho }g_{1}}{k_{1z}g_{2}},\\ B_{2}\left(k_{\rho }\right) & =i\frac{\varepsilon_{1}}{\varepsilon_{2}}\frac{2\mu_{1}k_{\rho }}{g_{2}},\\ C_{1}\left(k_{\rho }\right) & =i\frac{k_{1z}}{k_{\rho }}A_{1}\left(k_{\rho }\right)+i\frac{k_{1z}}{k_{\rho }}B_{1}\left(k_{\rho }\right),\\ C_{2}\left(k_{\rho }\right) & =i\frac{k_{1z}}{k_{\rho }}A_{2}\left(k_{\rho }\right)-i\frac{k_{2z}}{k_{\rho }}B_{2}\left(k_{\rho }\right). \end{array}$$

where the *f* and *g* coefficients are related to the Fresnel coefficients, specifically:

(C4)
$$\begin{array}{cc} f_{1}=\varepsilon_{2}k_{1z}-\varepsilon_{1}k_{2z}, & f_{2}=\varepsilon_{2}k_{1z}+\varepsilon_{1}k_{2z},\\ g_{1}=\mu_{2}k_{1z}-\mu_{1}k_{2z}, & g_{2}=\mu_{2}k_{1z}+\mu_{1}k_{2z}, \end{array}$$

Similar to the 2D Green’s function, the singular points in the 1D Green’s function occur when the denominators of Equations (C1) and (C2) equal zero, i.e., when *ρ* = 0, *k*_*ρ*_ = *k*_1_, or *k*_*ρ*_ = *k*_2_. The singular point *ρ* = 0 is independent of the integration variable *k*_*ρ*_ and is removable by using L’Hôpital’s rule. Considering only the reflected part of Equation (C1), and substituting the values of Bessel functions *J*_0_(0) = 1 and $\lim_{x\to 0}\frac{J_{1}\left(x\right)}{x}=\frac{1}{2}$ after applying L’Hôpital’s rule where necessary results in:

(C5)
$$\begin{array}{ll} E_{1\rho }^{V,\text{ref}}\left(\rho \to 0\right) & =0\\ E_{1\varphi }^{V,\text{ref}}\left(\rho \to 0\right) & =0\\ E_{1z}^{V,\text{ref}}\left(\rho \to 0\right) & =\frac{p_{z}}{4\pi \varepsilon_{0}\varepsilon_{1}}\overset{\infty }{\underset{0}{\int }}A_{1}k_{\rho }^{2}e^{\text{i}k_{1z}\left(z+z_{0}\right)}dk_{\rho }\\ E_{1\rho }^{H,\text{ref}}\left(\rho \to 0\right) & =cos\varphi \frac{p_{x}}{4\pi \varepsilon_{0}\varepsilon_{1}}\overset{\infty }{\underset{0}{\int }}e^{\text{i}k_{1z}\left(z+z_{0}\right)}\\ & \;\times \left[B_{1}\left(\frac{k_{\rho }^{2}}{2}+k_{1z}^{2}\right)+\frac{i}{2}C_{1}k_{\rho }k_{1z}\right]dk_{\rho }\\ E_{1\varphi }^{H,\text{ref}}\left(\rho \to 0\right) & =\sin\varphi \frac{p_{x}}{4\pi \varepsilon_{0}\varepsilon_{1}}\overset{\infty }{\underset{0}{\int }}e^{\text{i}k_{1z}\left(z+z_{0}\right)}\\ & \;\times \left[B_{1}\left(\frac{k_{\rho }^{2}}{2}-k_{1z}^{2}\right)-\frac{i}{2}C_{1}k_{\rho }k_{1z}\right]dk_{\rho }\\ E_{1z}^{H,\text{ref}}\left(\rho \to 0\right) & =0 \end{array}$$

Note that as *ρ* approaches 0, the directions of the cylindrical unit vectors depend on the chosen direction of approach. It is important to keep track of these directions when converting back to a Cartesian system.
